# Ginsenoside Rg3 Alleviates Cisplatin Resistance of Gastric Cancer Cells Through Inhibiting SOX2 and the PI3K/Akt/mTOR Signaling Axis by Up-Regulating miR-429

**DOI:** 10.3389/fgene.2022.823182

**Published:** 2022-03-03

**Authors:** Xiaofeng Wang, Rui He, Li Geng, Jing Yuan, Huijie Fan

**Affiliations:** Department of Medical Oncology, The First Affiliated Hospital of Zhengzhou University, Zhengzhou, China

**Keywords:** gastric cancer, cisplatin chemoresistance, ginsenoside Rg3, SOX2, miR-429, PI3K/Akt/mTOR signaling pathway

## Abstract

Platinum-based cytotoxic chemotherapy is considered the standard treatment for advanced gastric cancer (GC). However, cisplatin chemoresistance often occurs with the mechanisms being not well clarified, which results in the cancer recurrence and poor survival. Ginsenoside Rg3, isolated from the Chinese Herb Panax Ginseng, is recognized as an anti-cancer agent. Herein, we aimed to reveal whether Ginsenoside Rg3 alleviates cisplatin resistance and sensitizes GC cells to cisplatin-induced apoptosis, and draw out the underlying molecular mechanism in cisplatin-resistant GC cells. The lower expression of miR-429 was found in AGSR-CDDP cells; it was also in association with cisplatin-resistance in GC cells and expression of which was restored following Ginsenoside Rg3 treatment. We also demonstrated that miR-429 made a contribution toward chemosensitivity in GC cells partly through SOX2 regulation. SOX2 was found to contribute to developing platinum resistance and was an authentic target for miR-429 in AGSR-CDDP cells. Importantly, enforced expression of SOX2 with a pcDNA3-SOX2 construct lacking the 3′-UTR miRNA binding site diminished the cytotoxic effects of miR-429 in AGSR-CDDP cells. We demonstrated that Ginsenoside Rg3 enhanced chemosensitivity in AGSR-CDDP GC cells, at least in part, through up-regulating miR-429, thereby targeting SOX2 and modulating downstream PI3K/AKT/mTOR signaling. Ginsenoside Rg3 was also found to regulate apoptosis-related genes via miR-429 in cisplatin-resistant GC cells. Ginsenoside Rg3 treatment significantly suppressed the migration rate of AGSR-CDDP GC cells, while following transfection with anti-miR-429, the anti-migratory effects of Ginsenoside Rg3 was partially abolished. This data suggested that Ginsenoside Rg3 may impede the chemoresistance and migration of GC cells mainly mediated through miR-429. We concluded that miR-429-regulated SOX2 expression was one of the main mechanisms by which Ginsenoside Rg3 dramatically promoted its anticancer effects on cisplatin-resistant GC cells. We also underscored a supporting model in which miR-429 adjusted PI3K/AKT/mTOR signaling by regulating SOX2 in cisplatin-resistant GC cells.

## Introduction

Gastric cancer (GC) is considered as one of the most common causes of cancer-related deaths worldwide. The preferred option for the standard treatment of GC is surgical resection of the stomach; however, the majority of patients are often diagnosed at advanced stages when having surgery is not advised (Digklia and Wagner, 2016; Marin et al., 2016). Despite recent advances in the treatment of GC, chemotherapy is still one of the most important therapeutic options for advanced GC (He et al., 2017). Platinum-based cytotoxic chemotherapy such as cisplatin is considered as one of the preferred treatment options for advanced GC. Mechanically, cisplatin exerts its anti-cancer effects through the generation of DNA lesions followed by the activation of the DNA damage response and the induction of apoptosis (Galluzzi et al., 2012). Although the platinum agent cisplatin is the first-line chemotherapy for GC, the treatment success is severely limited due to the development of drug resistance (Wang et al., 2020). Hence, identifying pivotal genes and molecular pathways involved in GC progression and chemoresistance is important for developing effective therapeutic interventions against GC.

Aberrant activation of the PI3K/AKT signaling is of importance for chemoresistance and contributes to epithelial-mesenchymal transition (EMT) which occurs in metastatic and drug-resistant cancer cells (Fattahi et al., 2020). Sex-determining region Y-box 2 (SOX2) is a self-renewal transcription factor essential to maintain embryonic stem cells and cellular reprogramming; and contributes to tissue homeostasis and regeneration. Like many other developmental genes, SOX2 is abnormally expressed in a variety of human cancers and is involved in tumor progression and chemoresistance (Shen et al., 2017). Several lines of evidence have revealed the functional and reciprocal dependencies between SOX2 and the PI3K/AKT signaling pathway which may contribute to cancer metastasis and chemoresistance (Tripathi et al., 2017; Schaefer and Lengerke, 2020).

Aberrant expression and function of microRNAs (miRNAs, miRs) are strongly implicated in tumorigenesis. Moreover, the emerging role of miRNAs as potentially critical regulators involved in the development of chemoresistance highlights the significance of this class of non-coding RNAs in modulating drug resistance-related signaling pathways among human cancers including GC (Magee et al., 2015). Consistently, analyzing the expression profiling of miRNAs in the NCI-60 (a panel of 60 diverse human cancer cell lines) exposed to various chemical anti-cancer compounds revealed significant correlations between the miRNA expression profiles and drug potency patterns. These data indicated a role for such miRNAs in drug response and proposed that miRNAs may provide a substantial link for identifying mechanisms involved in chemoresistance in cancer cells (Blower et al., 2007).

Ginsenoside Rg3, one of the bioactive ginsenosides isolated from the Chinese Herb Panax Ginseng C.A. Meyer, is recognized as an antitumor agent for the prevention and treatment of cancers. The main mechanisms explaining the tumor inhibitory function of Ginsenoside Rg3 include the induction of apoptosis and the inhibition of proliferation, metastasis and angiogenesis, as well as the activation of immunity (Sun et al., 2017). Moreover, Ginsenoside Rg3 represents the potential to be used in combination with conventional chemotherapeutics, improving the efficacy and/or diminishing undesirable side effects through synergistic activities. The use of Ginsenoside Rg3 in combination cancer therapy may aid in the avoidance of toxicity and augmentation of chemosensitivity, even though the precise underlying molecular mechanisms remain to be further clarified (Sun et al., 2017; Nakhjavani et al., 2019).

In the present study, the functional role of Ginsenoside Rg3 in cisplatin-resistant GC cells was explored. We proposed that Ginsenoside Rg3 sensitized GC cells to cisplatin treatment through inhibiting SOX2 and the PI3K/AKT/mTOR signaling axis by up-regulating miR-429. Our study may pave the way toward understanding the platinum chemoresistance in GC and affirmed that Ginsenoside Rg3 could serve as a novel adjuvant agent to improve the chemosensitivity of GC to cisplatin.

## Materials and Methods

### Cells and Reagents

AGS GC cells and derived cisplatin-resistant cell line pair (AGSR-CDPP), which was obtained through a stepwise increasing drug doses method, were cultured in Dulbecco’s modified Eagle’s medium (DMEM) containing 10% FBS, 100 IU/ml of penicillin, and 100 µg of streptomycin (All from Gibco, MA, United States) at 37°C in a humidified atmosphere with 5% CO_2_. To maintain drug resistance, AGSR-CDDP cells were further incubated with 0.5 μg/ml of cisplatin. Ginsenoside 20(R)-Rg3 (Yuanye Inc., China) and cisplatin (CDDP, Sigma-Aldrich, United States) were dissolved in dimethyl sulfoxide (DMSO) and RPMI-1640, respectively. Both solutions were stored at a temperature of −20°C.

### MTT Assay

A tetrazolium-based colorimetric MTT assay was used to assess cell viability. The optical density of the DMSO-dissolved formazan salts was measured using a microtiter plate reader (BioTek, United States) at 490 nm.

### Apoptosis Assays

Annexin-V staining and propidium iodide (PI) exclusion were performed to indicate apoptosis using the Annexin-V FITC Apoptosis Detection Kit (Sigma) according to the manufacturer’s instructions. The Caspase-Glo 3/7 assay kit (Promega) was also used to evaluate the activity of caspase-3 and caspase-7 according to the manufacturer’s recommendation. A luminometer (Berthold, Germany) was used to measure the luminescence of each sample.

### Cell Migration Assay

Cell migration rate was determined using a wound-healing assay. Briefly, cells were seeded into 24-well plates and grown in FBS-free media. A plastic pipette tip was used to make a single vertical scratch wound on the monolayer of the cells. After washing with PBS, the debris or detached cells were removed and the results were photographed by an inverted microscope at 0 and 24 h.

### RNA Extraction, RT-qPCR, and TaqMan miRNA Array

TRIzol^®^ reagent (Invitrogen, Mulgrave, VIC, Australia) was employed to extract the total RNAs and the PrimeScript RT Master Mix (TAKARA) was used to synthesize cDNA. The real-time quantitative PCR (RT-qPCR) was performed on an ABI 7900 System (Applied Biosystems, CA, United States). Glyceraldehyde 3-phosphate dehydrogenase (GAPDH) was used to examine the relative expression of candidate genes. For determining amplification efficiencies, standard curves via plotting the logarithmic amount of five‐fold serially diluted cDNA input against the corresponding Ct values were drawn. The amplification efficiencies were calculated according to the slope of the standard curves and the following formula: E = 10^(−1/slope)^. The expression level of miR-429 was measured by specific primers and miRNA qPCR master mix kit (Stratagene). U48 snRNA was used to normalize the expression level of miR-429. Fold changes were calculated through the comparative threshold cycle (Ct) method. For expression profiling of miRNAs derived from three pairs of AGS and AGSR-CDDP cells, the RT-qPCR was performed by using gene-specific primers and TaqMan probes and TaqMan universal PCR master mix (PE Applied Biosystems).

### Transfection Assays

AGSR-CDDP cells were transfected with about 1 µg of pcDNA3-SOX2 (a SOX2 expression plasmid construct lacking the miR-429 binding site in the 3′-UTR) or the control empty vector using Lipofectamine 2000 (Invitrogen). Transfection of miR-429 mimics or negative control (25 nM); anti-miR-429 or scramble (50 nM); siRNA against SOX2 (si-SOX2) or negative control (50 nM) into GC cells was performed using Lipofectamine 2000 (Invitrogen). For luciferase reporter assay, GC cells were co-transfected with psi-CHECK2 luciferase reporter vector containing WT- or Mut-3′-UTR of SOX2 together with miR-429 mimics or the negative control. After 48 h, the relative luciferase activity was determined as the ratio of firefly to Renilla luciferase activity using the Dual-Luciferase Reporter Assay System (Promega).

### Western Blot Analysis

Protein lysates were isolated in radioimmunoprecipitation assay (RIPA) buffer. The BCA kit (Pierce, Thermo Fisher Scientific) was used to measure protein concentration. After separation on sulphate–polyacrylamide gel electrophoresis, the protein samples were transferred to PVDF membranes (BD Biosciences, Clayton, VIC, Australia). Membranes were incubated with primary antibodies (1:1,000) at 4°C overnight. The following monoclonal antibodies were used: anti-Bcl-2 (sc-7382), anti-Bax (sc-20067), and anti-p-Akt (sc-293125) (Santa Cruz Biotechnology). Secondary antibodies conjugated with the horseradish peroxidase enzyme were employed. The protein bands were visualized using an enhanced chemiluminescence kit (ECL, Amersham, United Kingdom) and the band intensities were analyzed using ImageJ.

### Statistical Analysis

The statistical analysis was accomplished by the Student’s t-test or two-way ANOVA using the Prism GraphPad 8.0 software. All data were presented as mean of three independent experiments ±standard deviation (SD). Differences were considered to be statistically significant when *p*-value < 0.05.

## Results

### Ginsenoside Rg3 Alleviated Cisplatin Resistance and Sensitized AGSR-CDDP GC Cells to Cisplatin-Induced Apoptosis

**FIGURE 1:**
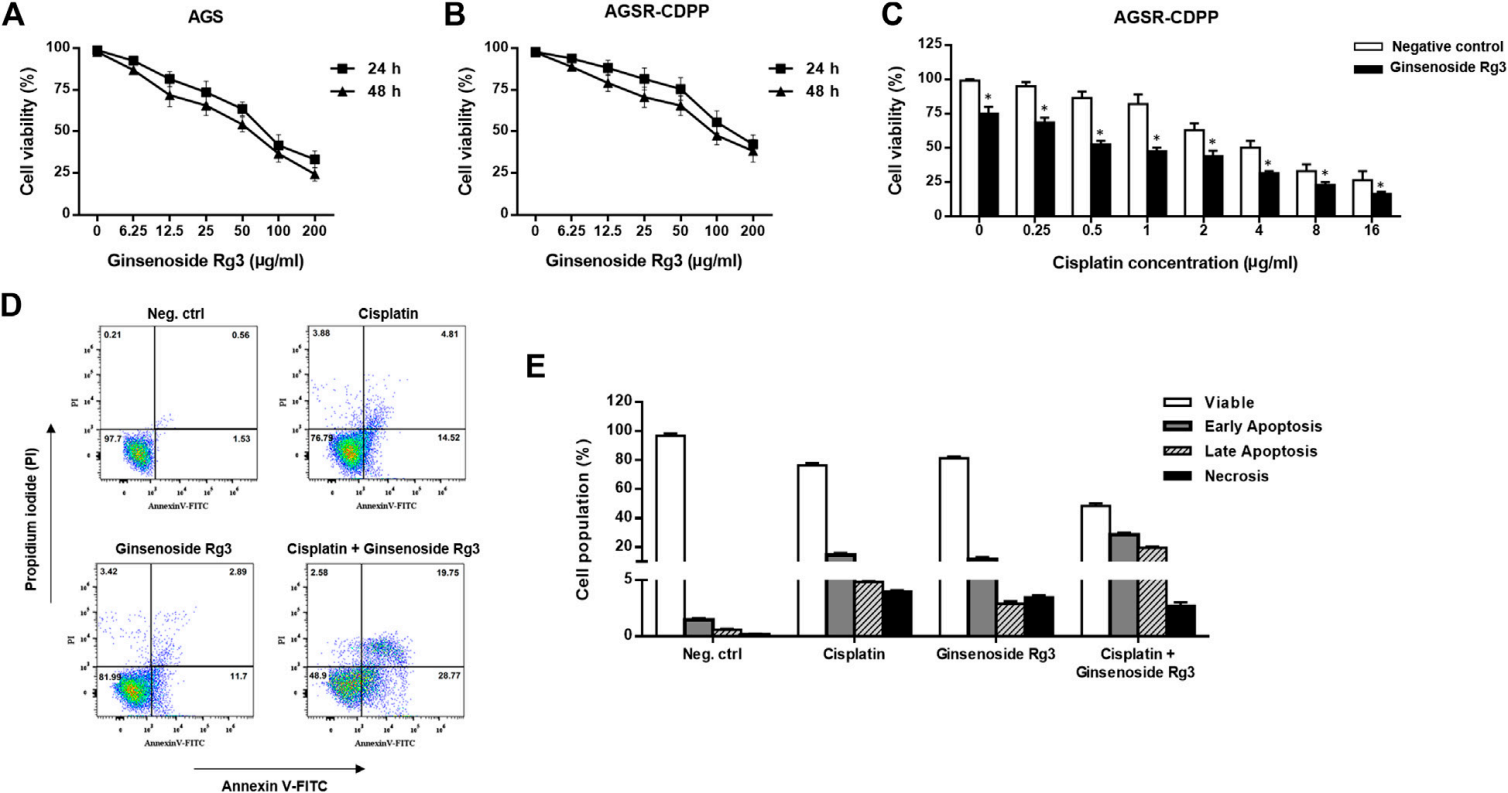
Ginsenoside Rg3 decreased cisplatin resistance and made AGSR-CDDP GC cells more sensitive to cisplatin-induced apoptosis. **(A,B)** Anti-proliferative effects of various concentrations of Ginsenoside Rg3 on AGS and AGSR-CDPP GC cells. The cytotoxic effects of Ginsenoside Rg3 on inhibiting cancer cell proliferation were similar between AGS and AGSR-CDDP cells. **(C)** Ginsenoside Rg3 enhanced the cytotoxic effect of different concentrations of cisplatin on AGSR-CDDP cells. Points/columns: the mean of three independent experiments; bars: standard deviations; * *p*-value < 0.05. **(D)** Effects of single and combination treatment with cisplatin and Ginsenoside Rg3 on inducing apoptosis in AGSR-CDDP cells. Flowcytometry analysis of AGSR-CDDP cells stained with Annexin V-FITC and PI staining revealed that both cisplatin and Ginsenoside Rg3 may trigger apoptosis (in comparison with the control cell group). However, as compared to either cisplatin or Ginsenoside Rg3, the combination treatment resulted in a markedly higher number of apoptotic cells. **(E)** The percentage of viable, early apoptotic, late apoptotic, and necrotic AGSR-CDDP cells following single or combination treatment with cisplatin and Ginsenoside Rg3.

Ginsenoside Rg3 was evaluated for cytotoxicity on cisplatin-resistant (AGSR-CDDP) and cisplatin-sensitive (AGS) GC cells at various concentrations (6.25, 12.5, 25, 50, 100, and 200 μg/ml) for 24 and 48 h. The MTT cell viability assay revealed that, whereas high concentrations of Ginsenoside Rg3 (100 and 200 μg/ml) decreased the viability of AGS and AGSR-CDDP cells, low concentrations of Ginsenoside Rg3 (6.25, 12.5, 25, and 50 μg/ml) had no significant toxicity effects after 24 and 48 h (Figures 1A,B). These observations showed that Ginsenoside Rg3 inhibited cell viability in both AGS and AGSR-CDPP cells in a comparable way. Accordingly, the half-maximal inhibitory concentration (IC50) value of Ginsenoside Rg3 was determined 50 μg/ml and was applied for the following experiments.

To demonstrate whether Ginsenoside Rg3 alleviate the resistance to cisplatin in GC cells, MTT assay was again exploited to assess the effects of the combination of Rg3 (50 μg/ml) and various concentrations of cisplatin (0.25, 0.5, 1, 2, 4, 8, 16 μg/ml) on the viability of AGSR-CDPP cells after 24 h. As shown in Figure 1C, we found that Ginsenoside Rg3 considerably enhanced the cisplatin sensitivity of AGSR-CDPP cells compared with cells incubated with cisplatin alone. Also, to evaluate the possibility of apoptosis induction, AGSR-CDPP cells were incubated with Ginsenoside Rg3 and cisplatin, alone or in combination, and induction of apoptosis was quantified following staining with Annexin-V-FITC and PI. Flow cytometry analysis of treated cells revealed that both Ginsenoside Rg3 and cisplatin-induced cell apoptosis compared to untreated control cells. Importantly, treatment of AGSR-CDPP cells with 50 μg/ml Ginsenoside Rg3 in a pre-treated manner (with 2 μg/ml cisplatin) promoted apoptosis in up to 48.52% of cells, which was divided into 28.77% and 19.75% early and late apoptosis, respectively (Figure 1D). Altogether, these observations proposed that Ginsenoside Rg3 could reduce cisplatin resistance and sensitize AGSR-CDPP cells to cisplatin-induced apoptosis.

### miR-429 is Down-Regulated in Cisplatin-Resistant GC Cells and Restored Following Ginsenoside Rg3 Treatment

**FIGURE 2:**
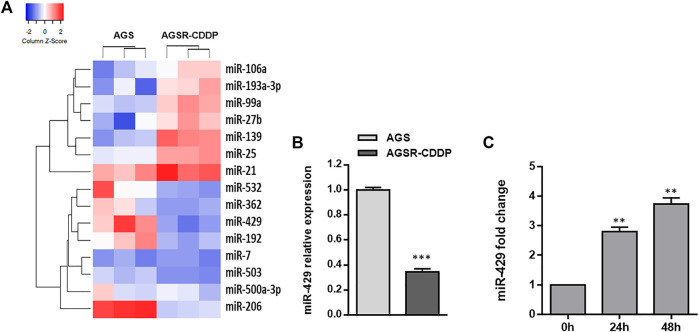
MiR-429 expression is reduced in cisplatin-resistant GC cells but restored after Ginsenoside Rg3 treatment. **(A)** Heatmap of the differentially expressed miRNAs obtained by TaqMan real-time PCR miRNA array in AGS GC cells and derived cisplatin-resistant cell line pair (AGSR-CDPP). The red showed a higher miRNA expression level and the blue indicated a lower miRNA expression level. The heatmap was drawn using Heatmapper (http://heatmapper.ca/expression/). **(B)** The relative miR-429 expression was determined by RT-qPCR in AGS and AGSR-CDPP GC cells. U48 snRNA was used as an internal reference. **(C)** Ginsenoside Rg3 caused a significant increase of miR-429 expression in AGSR-CDDP GC cells in a time-dependent manner. Columns: the mean of three independent experiments; bars: standard deviations; ** *p*-value < 0.01, *** *p*-value < 0.001.

As mounting evidence suggests the contribution of miRNAs in the development of drug resistance in cancer cells, we exploited a TaqMan real-time PCR miRNA array to identify the level of miRNAs differentially expressed between cisplatin-resistant (AGSR-CDDP) and cisplatin-sensitive (AGS) GC cells. To this end, we evaluated the expression levels of 50 candidate miRNAs in AGS and AGSR-CDDP GC cells. The results revealed 15 miRNAs that differentially expressed—including seven up-regulated miRNAs, while eight down-regulated miRNAs—in AGSR-CDDP GC cells relative to the parental sensitive cells (Figure 2A). Among miRNAs differentially expressed, miR-429 was found to be lowered in AGSR-CDDP GC cells and selected for further analysis to determine its function in the cisplatin resistance in GC cells. To answer this question that what are the potential effects of miR-429 in the cisplatin resistance of GC cells, the expression of miR-429 in AGS and AGSR-CDPP cells was also measured by RT-qPCR. Data elucidated that miR-429 expression was lower in cisplatin-resistant AGSR-CDPP GC cells than parental sensitive AGS cells (*p*-value <0.001; Figure 2B). These data suggested that the decreased expression level of miR-429 may be correlated with cisplatin resistance in GC cells. In order to find out the possibility of anti-cancer effects of Ginsenoside Rg3 through regulating miR-429, the expression status of this miRNA in AGSR-CDDP that were pre-incubated with or without Ginsenoside Rg3 was measured. RT-qPCR results showed that miR-429 was up-regulated in 50 μg/ml Ginsenoside Rg3-incubated AGSR-CDDP cells, in comparison to the control cells (Figure 2C). These findings suggested that Ginsenoside Rg3 may exert its anti-cancer effects through up-regulating miR-429 in AGSR-CDDP GC cells.

### miR-429 Functionally Targets and Suppresses SOX2

**FIGURE 3:**
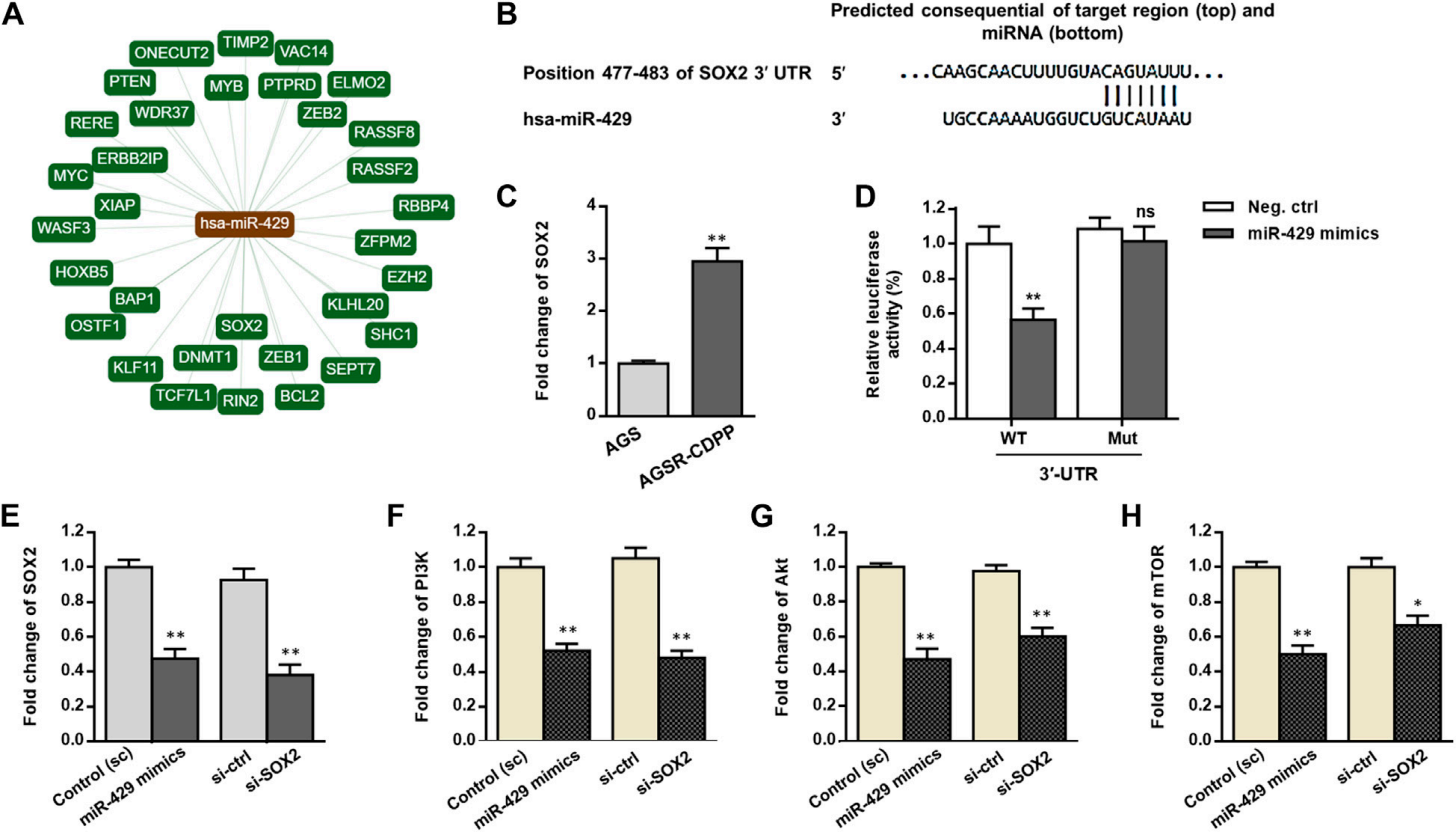
miR-429 may adjust the PI3K/AKT/mTOR signaling by regulating SOX2. **(A)** The network of interactions between miR-429 and its verified downstream target transcripts provided by miRTargetLink Human algorithm (https://ccb-web.cs.uni-saarland.de/mirtargetlink). **(B)** The specific miR-429 binding site in the 3′-UTR of SOX2 predicted by TargetScan (http://www.targetscan.org). **(C)** SOX2 transcript levels, assessed by RT-qPCR, in AGS and AGSR-CDPP GC cells. Results showed that SOX2 transcript levels were much greater in AGSR-CDPP GC cells than AGS cells. **(D)** Luciferase activity in AGSR-CDPP GC cells 28 h after co-transfection with SOX2-3′UTR luciferase reporter construct and miR-429 mimics or negative control. miR-429 suppressed the luciferase activity in the cells transfected by wild type (WT) SOX2-3′UTR luciferase construct (*p* < 0.01); however, it had no significant effect in the cells transfected with mutated (Mut) SOX2-3′-UTR construct., suggesting that SOX2 is a direct target of miR-429. **(E–H)** The expression of SOX2 and the PI3K/Akt/mTOR axis at transcript levels measured by RT-qPCR in AGSR-CDPP cells that were transfected with either miR-429 mimics or siRNAs against SOX2 (si-SOX2) compared to the corresponding controls. Transfection of miR-429 mimics had comparable effects with the siRNA-knockdown of SOX2 and down-regulated the expression levels of SOX2 and the PI3K/Akt/mTOR axis in AGSR-CDPP cells. Columns, mean of three different experiments; bars, SD. * *p*-value < 0.05, ** *p*-value < 0.01, ns: not significant.

We used the bioinformatics analysis tools including miRTargetLink Human and TargetScan to identify potential targets of miR-429 (Figures 3A,B). Among several transcripts with conserved sites, we focused on SOX2 because it was previously found as a master regulator for gastric tumorigenicity and chemoresistance (Tian et al., 2012; Hütz et al., 2014). We first determined and compared the expression levels of SOX2 in the AGS GC cell line and its resistant derivative (AGSR-CDDP). As demonstrated in Figure 3C, the expression level of SOX2 in AGSR-CDDP cells was markedly higher than AGS cells (*p* < 0.001). Regarding the substantially reduced expression of miR-429 in AGSR-CDDP cells, it is fair to draw a conclusion that there may be an association between miR-429 and SOX2 in cisplatin-resistant GC cells. To further confirm whether miR-429 directly binds to and targets SOX2 in GC cells, the luciferase reporter constructs containing the Renilla luciferase encoding gene fused to either 3′-UTR of SOX2 that contained miR-429 binding site (WT) or mutated targeting site (Mut) was used. We found that miR-429 specifically suppressed the luciferase activity of a reporter that included the 3′UTR of WT SOX2 in GC cells (Figure 3D). This suppression was particular to the predicted miR-429 target sites, as no significant change was detected in the relative luciferase activity of the Mut 3′UTR SOX2 reporter. These findings fruitfully highlighted that miR-429 is capable of binding to the specific target sites that were located within 3′-UTR of SOX2 mRNA.

To answer this question that how miR-429 affects the SOX2 expression level in GC cells, we transfected AGSR-CDDP cells with either miR-429 mimics or scramble, and the mRNA levels of SOX2 were analyzed. Compatible with the results of the reporter assay, by the time miR-429 mimics were transfected into GC cells, SOX2 transcripts were considerably reduced (*p* < 0.01; Figure 3E). Consistently, by using siRNA that was specifically designed for SOX2 (si-SOX2), SOX2 mRNA levels were decreased (*p* < 0.01, Figure 3F).

In addition, to further understand the functional importance of SOX2, we measured the transcript levels of PI3K/AKT/mTOR signaling after transfecting the GC cells using miR-429 mimics. RT-qPCR analysis revealed that a considerable reduction in mRNA levels of PI3K/AKT/mTOR signaling axis in AGSR-CDDP cells that were transfected with miR-429 mimics than the cells that were transfected with scramble (Figure 3G). The same findings were also obtained when siRNA against SOX2 was used (Figures 3E–H). Overall, these findings attributed a regulatory function to SOX2 on downstream PI3K/AKT/mTOR signaling; we also highlighted a supporting model in which miR-429 modulated the PI3K/AKT/mTOR signaling by regulating SOX2.

### miR-429 Makes a Contribution Toward Chemosensitivity in GC Cells Partly Through Regulation of SOX2

**FIGURE 4:**
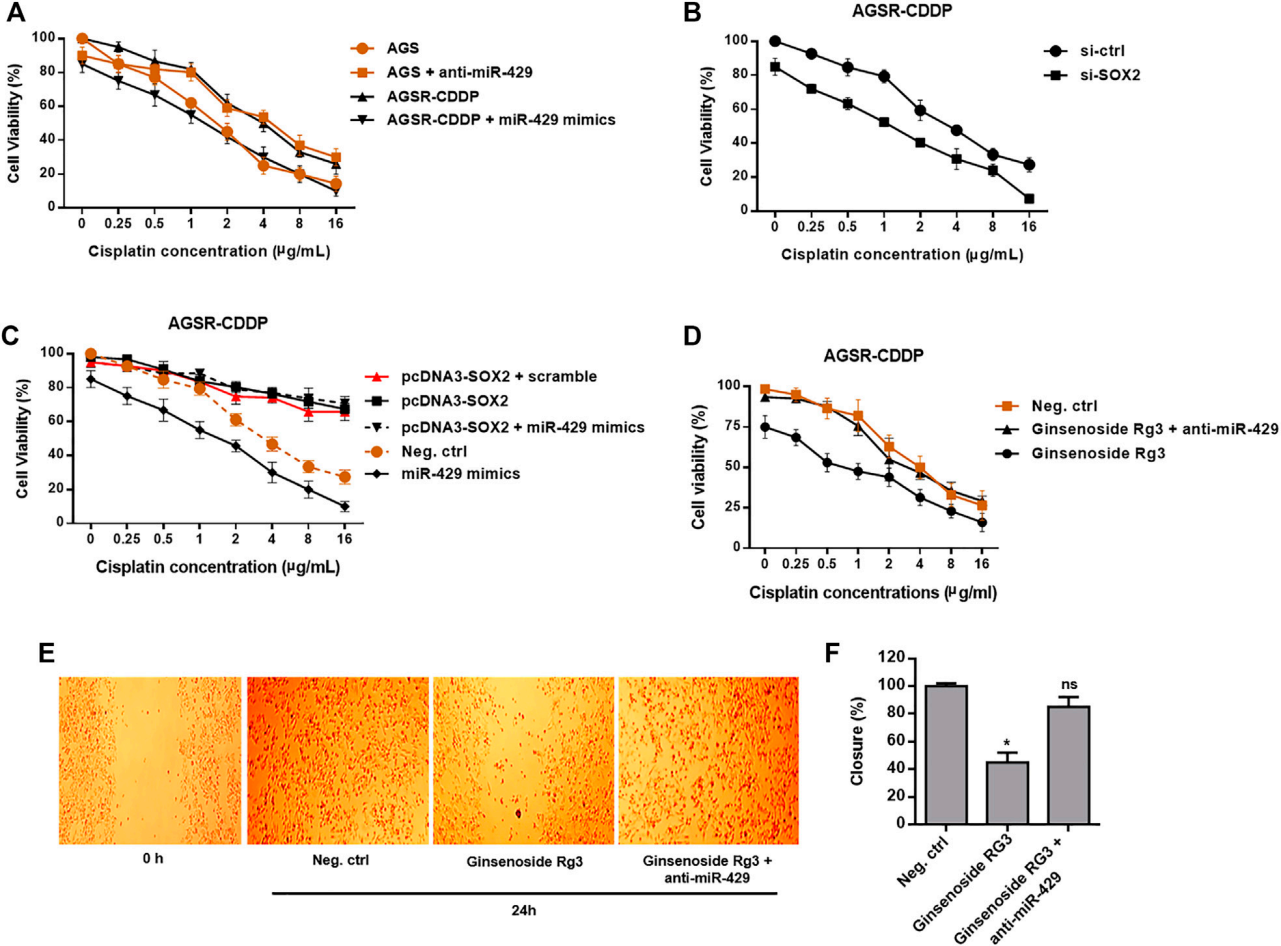
Ginsenoside Rg3 inhibits chemoresistance and migration of GC cells through the functional regulatory role of miR-429. **(A)** After 24 h of transfection, miR-429 mimics significantly enhanced the cisplatin sensitivity of AGSR-CDDP cells. Conversely, AGS cells transfected with anti-miR-429 were less sensitive to cisplatin than negative control cells, proposing that miR-429 contributes to chemosensitivity in GC cells. **(B)** siRNA knockdown of SOX2 resulted in a dramatic increase in the cisplatin sensitivity of AGSR-CDDP cells, compared to the negative control cells, verifying that SOX2 made a contribution to developing cisplatin resistance in GC cells. **(C)** Enforced expression of SOX2 significantly enhanced the cisplatin resistance of AGSR-CDDP cells in comparison with the negative control cells. Importantly, transfection of pcDNA3-SOX2 construct lacking the miR-429 target site in the 3′-UTR rescued AGSR-CDDP cells from the cytotoxic effects of miR-429. **(D)** Ginsenoside Rg3 significantly enhanced the cisplatin resistance of AGSR-CDDP cells in comparison with the negative control cells. Importantly, transfection of anti-miR-429 diminished the cytotoxic effects of Ginsenoside Rg3, implying that the cytotoxic effects of Ginsenoside Rg3 on AGSR-CDDP cells might be related to the up-regulation and consequent function of miR-429. Points: the mean of three independent experiments; bars: standard deviations. **(E,F)** Ginsenoside Rg3 inhibited the migration rate of AGSR-CDDP cells considerably more than the negative control cells. Importantly, transfection of anti-miR-429 reduced the anti-migratory activity of Ginsenoside Rg3, implying that the inhibitory effects of Ginsenoside Rg3 on AGSR-CDDP cell migration might be attributed to the up-regulation and consequent function of miR-429. Photomicrographs are representative of at least three independent experiments. Cell migration was measured by comparing cell-covered areas at the indicated time points according to image analysis. Columns, mean of three different experiments; bars, SD. * *p*-value < 0.05, ns: not significant.

To uncover whether miR-429 promotes chemosensitivity of AGSR-CDDP cells to cisplatin, we restored miR-429 expression in AGSR-CDDP cells—a process that was accomplished by transfecting the cisplatin-resistant cells with miR-429 mimics. Then, using miR-429 antisense-oligonucleotides (anti-miR-429), we knocked down miR-429 expression in AGS cells. The overexpression and interference effects were confirmed by RT-qPCR (Supplementary Figure S1). As expected, we found that the restoration of miR-429 considerably enhanced cisplatin sensitivity of AGSR-CDDP cells after 24 h of treatment. Meanwhile, the sensitivity of AGS cells—that were transfected with anti-miR-429— to cisplatin was decreased than the negative control cells (Figure 4A). Importantly, MTT assay confirmed that siRNA knockdown of SOX2 in AGSR-CDDP cells—that were previously incubated with a divagated concentration of cisplatin for 24 h—significantly enhanced the cisplatin sensitivity of AGSR-CDDP cells in comparison to the negative control cells (Figure 4B), verifying that SOX2 makes a contribution to developing cisplatin resistance in GC cells.

According to the accumulating evidence delineating that miR-429 has several potential targets, so we hypothesized that its effects on the cisplatin resistance could be somehow attributed to the targeting of SOX2. To this end, we transfected the pcDNA3-SOX2 construct lacking the miR-429 target site in the 3′-UTR into AGSR-CDDP cells. The increased levels of SOX2 transcript were the main findings of this process, which in turn significantly enhanced the cisplatin resistance of AGSR-CDDP cells in comparison with the negative control cells (Figure 4C). Besides, in order to investigate whether enforced expression of SOX2 could rescue AGSR-CDDP cells from the cytotoxic effects of miR-429 or not, AGSR-CDDP cells were co-transfected with pcDNA3-SOX2 and miR-429 mimics. Our findings revealed that the cisplatin resistance of AGSR-CDDP cells co-transfected with pcDNA3-SOX2 and miR-429 mimics did not significantly differ from those cells that were transfected with pcDNA3-SOX2. However, the cell viability rate of these cells was significantly greater than the negative control cells or the cells transfected with miR-429 mimics (Figure 4C). This can *ipso facto* indicate that the enforced expression of SOX2 (with a plasmid construct without 3′-UTR miRNA binding site) can diminish the cytotoxic effects of miR-429 in AGSR-CDDP cells. Taken together, these data suggested that expression status of miR-429 affects chemosensitivity in GC cells and highlighted that the cytotoxic effects of miR-429 can be imputed to being targeted of SOX2 in cisplatin-resistant AGSR-CDDP cells.

### Ginsenoside Rg3 Inhibits Chemoresistance and Migration of GC Cells Through Mediating miR-429

According to Figure 1C, we observed an increase in the expression level of miR-429 when AGSR-CDDP cells were incubated with Ginsenoside Rg3. To further confirm the regulatory mechanism whereby Ginsenoside Rg3 restrains cisplatin resistance in AGSR-CDDP GC cells, Ginsenoside Rg3-pre-treated AGSR-CDDP cells were simultaneously transfected with anti-miR-429 in the presence of a divagated concentration of cisplatin for 24 h. Accordingly, MTT cell viability assay showed that in comparison to the control cells, the suppression of miR-429 can considerably diminish the suppressive impressions of Ginsenoside Rg3 on cisplatin chemoresistance in AGSR-CDDP cells (Figure 4D), suggesting that the cytotoxic effects of Ginsenoside Rg3 on AGSR-CDDP cells might be attributed to the up-regulation and consequent function of miR-429.

Moreover, to determine whether the migration capability of AGSR-CDDP cells was affected by Ginsenoside Rg3, a wound-healing assay was exploited. To this end, AGSR-CDDP cells were incubated with Ginsenoside Rg3 (50 μg/ml) and analysis of the cell-covered areas at the time of wounding (0 h) and 24 h thereafter indicated that Ginsenoside Rg3-treated AGSR-CDDP cells unveiled a lower capacity to migrate than control cells after 24 h. Interestingly, transfection of anti-miR-429 into AGSR-CDDP cells reduced the suppressive outcomes of Ginsenoside Rg3 on the migration rate of GC cells and consequentially extricated AGSR-CDDP cells from the anti-migration effects of Ginsenoside Rg3 (Figure 4E,F). These data can *ipso facto* show that Ginsenoside Rg3 may impede the GC cell migration mainly through inducing the expression of miR-429.

### Ginsenoside Rg3 Inhibits SOX2 and the PI3K/AKT/mTOR Signaling Axis *via* miR-429 in Cisplatin-Resistant GC Cells

**FIGURE 5:**
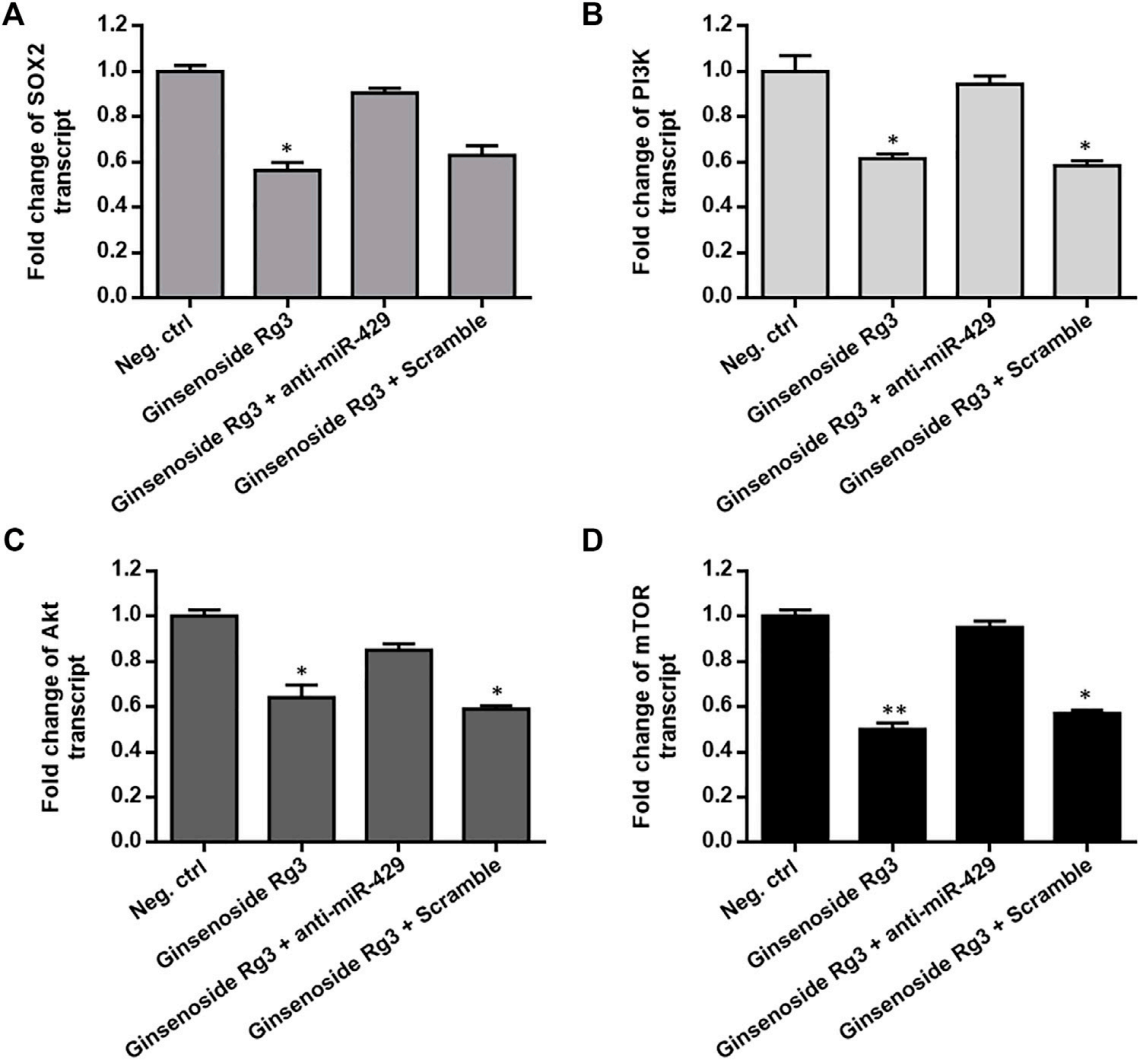
Ginsenoside Rg3 suppresses the expression of SOX2 and the PI3K/AKT/mTOR signaling axis in cisplatin-resistant GC cells through miR-429. Ginsenoside Rg3 treatment dramatically reduced the transcript levels of SOX2 **(A)** and PI3K/AKT/mTOR axis **(B–D)** as measured by RT-qPCR. However, the inhibitory effect of Ginsenoside Rg3 was partially diminished after anti-miR-429 transfection compared to cells transfected with the scramble. GAPDH was used as a housekeeping gene. Columns: the mean of three independent experiments; bars: standard deviations; * *p*-value < 0.05, ** *p*-value < 0.01.

The functional role of PI3K/AKT/mTOR signaling pathway in tumor progression and resistance to chemotherapeutic agents and the interconnectivity between SOX2 and PI3K/AKT have been previously proposed (Yang et al., 2014; Garros-Regulez et al., 2016). To assess whether Ginsenoside Rg3 inhibited the expression of SOX2 and the PI3K/AKT/mTOR signaling axis in cisplatin-resistant GC cells, AGSR-CDDP cells were incubated with Ginsenoside Rg3 and the transcript levels of SOX2 and the PI3K/AKT/mTOR signaling axis were determined by RT-qPCR. As illustrated in Figure 5, the transcript levels of SOX2 and PI3K/AKT/mTOR axis were significantly decreased by Ginsenoside Rg3 treatment. To explore the regulatory mechanism whereby Ginsenoside Rg3 restrains SOX2 and PI3K/AKT/mTOR axis, AGSR-CDDP cells were incubated with 50 μg/ml Ginsenoside Rg3 and simultaneously transfected by anti-miR-429 or negative control scramble. RT-qPCR results indicated that the inhibitory effect of Ginsenoside Rg3 was diminished following anti-miR-429 transfection than the cells that were transfected with scramble (Figures 5A–D). Additionally, western blot analysis showed that p-AKT level was considerably decreased in Ginsenoside Rg3-treated cells compared to the negative control. Results also showed that the inhibitory effect of Ginsenoside Rg3 on phosphorylated levels of AKT was reduced when anti-miR-429 was transfected to the cells (Supplementary Figure S2). Altogether, these data highlighted that Ginsenoside Rg3 inhibited SOX2 and the PI3K/AKT/mTOR signaling via miR-429 in cisplatin-resistant GC cells.

### Ginsenoside Rg3 Regulates Apoptosis-Related Genes *via* miR-429 in Cisplatin-Resistant GC Cells

**FIGURE 6:**
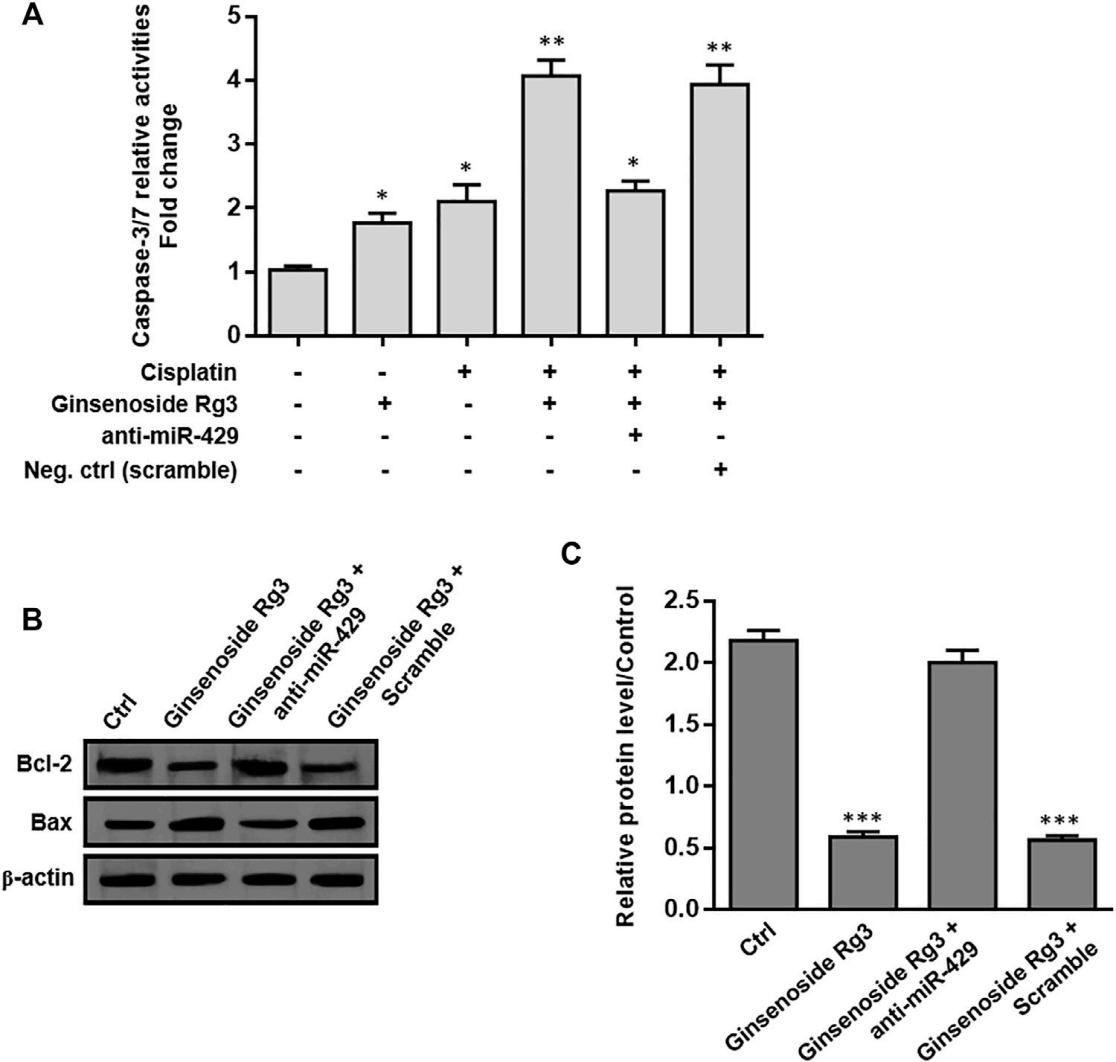
Ginsenoside Rg3 regulates apoptosis-related genes in cisplatin-resistant GC cells through miR-429. **(A)** Treatment of Ginsenoside Rg3 or cisplatin for 48 h caused an increase in caspase-3/7 activity in AGSR-CDPP cells. Importantly, Ginsenoside Rg3 synergistically increased cisplatin-induced caspase-3/7 activity, which was reduced after anti-miR-429 transfection. **(B)** Western blot analysis demonstrated an up-regulation in the expression level of the anti-apoptotic protein Bcl-2 and a down-regulation in the expression level of the pro-apoptotic protein Bax, following 48 h after treatment in AGSR-CDDP cells. Importantly, the effects of Ginsenoside Rg3 on the protein levels of these apoptosis-related genes were reversed by anti-miR-429 transfection compared to those cells transfected with scramble. Western blot images were representative of at least three independent experiments. **(C)** The Bcl-2 to Bax ratio was significantly reduced in Ginsenoside Rg3-incubated GC cells which were pre-treated with cisplatin. The ratio of Bcl-2 to Bax was reversed when Ginsenoside Rg3-incubated AGSR-CDDP cells were transfected with anti-miR-429. The density of bands was measured by ImageJ software and represented as a relative intensity. Columns, mean of three different experiments; bars, SD. * *p*-value < 0.05, ** *p*-value < 0.01, *** *p*-value < 0.001.

To evaluate the effect of Ginsenoside Rg3 on apoptosis-related genes, we first measured the caspase-3/7 activity assay by colorimetric method and the protein levels of Bax and Bcl-2 were determined by western blotting. As illustrated in Figure 6A, caspase-3/7 activity was induced by Ginsenoside Rg3 or cisplatin treatment after 48 h. Ginsenoside Rg3 also enhanced the cisplatin-induced caspase-3/7 activity, which was diminished following anti-miR-429 transfection. Furthermore, we demonstrated that Ginsenoside Rg3 incubation in AGSR-CDDP cells which were pre-treated with cisplatin caused a significant augment in the expression level of the pro-apoptotic protein Bax. In contrast, the expression level of the anti-apoptotic protein Bcl-2 was decreased in the Ginsenoside Rg3-treated cell group after 48 h (Figure 6B). The results also indicated that the ratio of Bcl-2 to Bax was considerably lowered in Ginsenoside Rg3-stimulated GC cells which were pre-treated with cisplatin (*p* < 0.001, Figure 6C), indicating the apoptotic effects of Ginsenoside Rg3 on cisplatin-resistant GC cells. Importantly, Ginsenoside Rg3 was found to enhance the effect of cisplatin on the expression of apoptosis-related genes, which could be reversed by anti-miR-429 transfection (Figures 6A–C). The results proposed that Ginsenoside Rg3 regulated apoptosis-related genes via miR-429 in cisplatin-resistant GC cells.

## Discussion

The therapeutic efficacy of platinum-based cytotoxic chemotherapy regimens is restricted, as evidenced by the occurrence of chemoresistance, which is accompanied by disease recurrence and leads to GC treatment failure (Orditura et al., 2014). Albeit the decreased drug uptake, enhanced drug efflux, increased drug metabolism, modified molecular drug targets and attenuation of DNA damage-mediated apoptotic signals may be possible explanations for cisplatin resistance (Siddik, 2003; Galluzzi et al., 2012), the precise mechanism underlying the cisplatin-induced resistance in GC is believed to be complex and still not clearly clarified. Accordingly, identifying pivotal genes and understanding the molecular pathways involved in GC progression and chemoresistance is important for developing therapeutic intervention strategies against GC. Recent studies have also revealed that SOX2 makes a contribution to chemoresistance and promotes EMT in different types of human cancers (Cui et al., 2020; Zhu et al., 2021a; Zhu et al., 2021b). However, the underlying molecular regulatory mechanism of this event remains to be further elucidated. It was shown that overexpression of SOX2 is accompanied by the resistance of prostate cancer cells to the chemotherapeutic agent paclitaxel. Also, because the chemoresistance effect of Sox2 is mediated by hyperactivation of the PI3K/Akt signaling, it appears that targeted therapy against Sox2 administered in combination with paclitaxel may be a promising therapy in chemoresistant prostate cancer (Li et al., 2014). Recently, it was demonstrated that the PI3K/AKT/SOX2 axis has a significant role in the development of R-CHOP resistance in the proportion of cancer stem-like cells (CSCs) in diffuse large B cell lymphoma. Importantly, when combined with the R-CHOP regimen, the PI3K/AKT inhibitor converted CSCs to differentiated cancer cells by destabilizing SOX2 level, thus inhibiting the growth of resistant cells that were highly sensitive to the R-CHOP regimen (Chen et al., 2020).

Altered expression of miRNAs is a common characteristic of human cancers and may render cancer cells resistant to chemotherapeutic agents (Si et al., 2019). The recent attention focused on miRNAs has resulted in a better understanding of the mechanisms through which GC cells develop therapeutic resistance characteristics (Poursheikhani et al., 2020). Apoptosis inhibition is one of the important underlying mechanisms that contribute to cisplatin resistance (Luo et al., 2019). Mounting evidence has demonstrated that miRNAs make a contribution to cisplatin resistance through modulating apoptosis-related signaling. For example, up-regulation of miR-193a-3p was found to be correlated with the development of cisplatin resistance through regulation of the mitochondrial apoptosis pathway in CD44^+^ GC cells (Lee et al., 2019). The Forkhead box O3a (FOXO3a) is recognized as an important transcriptional regulator which regulates a network of genes involved in several cellular processes, including cell cycle progression, apoptosis, and autophagy (Nho and Hergert, 2014). The functional outcome of these FOXO3a-regulated processes may lead to the drug-resistance phenotype. He et al. (2017) indicated up-regulation of miR-25 was essential for GC cells to establish a cisplatin-resistant phenotype through a FOXO3a-dependent mechanism. Ge et al. (2016) investigated the roles of miR-421, a HIF-1*α* induced miRNA detected to be higher in advanced GC, and found that overexpression of miR-421 enhanced metastasis, suppressed apoptosis, and induced cisplatin resistance by targeting E-cadherin and caspase-3 in GC cells. It was demonstrated that Calpain1 and Calpain2, regulated by Calpain small subunit 1 (CAPNS1), were implicated in cisplatin resistance in GC by cleaving the downstream proteins caspase3 and PARP1 to induce apoptosis. Wang and Ji (2018) reported that down-regulation of miR-17-5p sensitized GC cells to cisplatin-induced apoptosis, at least partially via targeting p21. It was also shown that miR-99a and miR-491 were up-regulated in resistant GC cells and take a role in cisplatin resistance through targeting CAPNS1-associated pathway in GC cells (Zhang et al., 2016). Low expression of miR-524-5p was found to be associated with lymph node metastasis and advanced TNM stage. Up-regulation of miR-524-5p increased the cisplatin sensitivity of GC cells by modulating proliferation and metastasis through targeting SOX9 (Wang et al., 2017).

Herein, we identified miR-429 to be down-regulated in AGSR-CDDP GC cells in comparison to the parental AGS cells (Figures 2A,B), so we investigated the possibility of the contribution of this miRNA in cisplatin-resistance of GC cells. Additionally, among different potential targets for miR-429, we focused on SOX2 because it has been reported to be an important contributor to chemoresistance due to its high expression in GC cells (Figure 3C). Our data revealed that the enforced expression of miR-429 in AGSR-CDDP GC cells could enhance the cisplatin sensitivity, while miR-429 knockdown in AGS cells diminished the sensitivity to cisplatin (Figure 4A). As we identified that SOX2, as a direct and authentic target of miR-429, made a contribution to developing cisplatin resistance (Figure 4B), we sought to investigate whether the inhibitory effects of miR-429 on the cisplatin resistance could be somehow attributed to the targeting SOX2. Remarkably, we showed that enforced expression of SOX2 (induced using a plasmid lacking 3′-UTR miRNA target site) can rescue AGSR-CDDP cells from the cytotoxic effects of miR-429 (Figure 4C). These findings led us to propose that the cytotoxic effects of miR-429 might be imputed to the down-regulation of SOX2 in AGSR-CDDP GC cells.

There is increasing evidence indicating that phytochemicals show great roles in the prevention and treatment of cancer through their modulatory effects on non-coding RNAs (Ruiz‐Manriquez et al., 2021). Curcumin, a major phytochemical in turmeric, has been shown to have an important role in inhibiting proliferation of solid cancer cells. It was shown that curcumin inhibited the proliferation of GC cells by down-regulating the c-Myc/H19 lncRNA pathway (Liu et al., 2016). Liu et al. (2018) suggested that curcumin enhanced the activity of PTEN by down-regulating miR-21 and prevented the activity of the PI3K/AKT signaling which in turn inhibited the biological activity of GC cells. Ginsenoside Rg3, a steroidal saponin derived from Panax Ginseng in traditional Chinese medicine, has been found to have anti-tumor activity including promoting cytotoxicity of chemotherapy in various types of human cancers (Tian et al., 2016; Yuan et al., 2017; Peng et al., 2020). Ginsenoside Rg3 can enhance the sensitivity to chemotherapy when used in combination with cisplatin (Lee et al., 2014) or docetaxel (Kim et al., 2010). Li and Qi (2019) illustrated that Ginsenoside Rg3 inhibited migration and invasion, and promoted apoptosis of colorectal cancer cells by suppression expression of LncRNA CCAT1. Apart from the anti-cancer activity, Ginsenoside Rg3 is recognized to boost immune response. It was demonstrated that Ginsenoside Rg3 attenuated cisplatin resistance in lung cancer through down-regulating PD-L1 and resuming immune. However, whether Rg3 prevents chemoresistance by regulating PD-L1 mediated immune escape needs to be further clarified. It was thought that interaction between the PD-L1 on cancer cells with the PD-1 receptor on cytotoxic T lymphocytes resulted in cancer immune escape. As a possible mechanism, Ginsenoside Rg3 may enhance the immune cytotoxity of T cells by targeting PD-L1 induced by chemotherapy (Jiang et al., 2017). Regarding increasing attention has accordingly been paid to natural products, we considered whether we could use Ginsenoside Rg3 in inducing chemosensitivity in GC cells. In our study, we investigated the ability of Rg3 to trigger apoptosis of GC cells alone or in combination with cisplatin treatment. We found that the cytotoxic effects of Ginsenoside Rg3 on inhibiting cell growth were comparable between AGS and AGSR-CDDP cells. However, as compared to either cisplatin or Ginsenoside Rg3, the combination treatment caused a significantly higher number of apoptotic cells in cisplatin-resistant GC cells (Figure 1).

**FIGURE 7:**
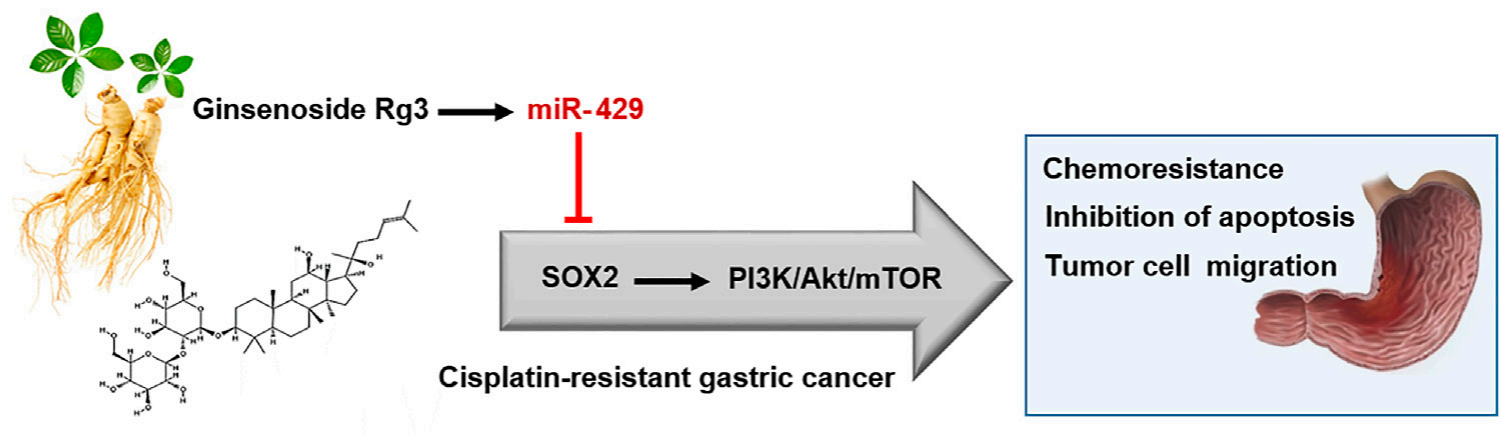
A schematic model illustrating how Ginsenoside Rg3 inhibits chemoresistance and migration of GC cells through the functional regulatory role of miR-429. miR-429 contributes to chemosensitivity in GC cells partly through regulating SOX2 and the PI3K/AKT/mTOR signaling axis in cisplatin-resistant GC cells. Modulation of SOX2 *via* miR-429 may be one of the main mechanisms by which Ginsenoside Rg3 exerts.

Despite the fact that Ginsenoside Rg3 has an anti-tumor effect on GC, the underlying molecular mechanisms by which Ginsenoside Rg3 may sensitize GC cells to cisplatin resistance are not clearly characterized. Our data showed that the decreased level of miR-429 was restored following Ginsenoside Rg3 treatment (Figure 2C). One of the reasons for the regulation of miR-429 by Ginsenoside Rg3 could be the molecular effects of this steroidal saponin on GC cells, especially in term of epigenetic alterations. Furthermore, we revealed that Ginsenoside Rg3 reduced chemoresistance and migration of GC cells via the functional regulatory role of miR-429 (Figure 4). Accordingly, we demonstrated that Ginsenoside Rg3 may adjust the expression of SOX2 and the PI3K/AKT/mTOR signaling axis by up-regulating miR-429 in cisplatin-resistant GC cells (Figure 5). Mounting evidence suggests that SOX2 inhibits apoptosis and subsequently drives chemoresistance in various types of human cancers (Zhang et al., 2020). Consistent with these studies, we found that Ginsenoside Rg3 regulated apoptosis-related genes through up-regulating miR-429 (Figure 6) in cisplatin-resistant GC cells. To the best of our knowledge, this study is the first to show that Ginsenoside Rg3 may exert its anti-cancer effects through up-regulating miR-429 in AGSR-CDDP GC cells. We proposed a model in which up-regulation of miR-429 in Ginsenoside Rg3-AGSR-CDDP cells may alleviate cisplatin resistance of GC cells via modulating SOX2 and the PI3K/Akt/mTOR signaling axis (Figure 7).

In conclusion, the occurrence of cisplatin-associated chemoresistance highlights the need for developing alternative therapeutics which can be used alone or in combination with conventional chemotherapeutic agents such as cisplatin against GC. Ginsenoside Rg3, when combined with cisplatin, considerably improved the chemosensitivity of GC cells. The cytotoxic effects of Ginsenoside Rg3 on cisplatin-resistant GC cells might be attributed to the up-regulation of miR-429. The functional regulatory role of miR-429 in regulating SOX2 and downstream PI3K/AKT/mTOR signaling may be a possible underlying mechanism.

## Data Availability

Data supporting the findings of this study are available from the corresponding author upon reasonable request.
