# Supplementary material for: Ginsenoside Rg3 Alleviates Cisplatin Resistance of Gastric Cancer Cells Through Inhibiting SOX2 and the PI3K/Akt/mTOR Signaling Axis by Up-Regulating miR-429
Source: Front Genet. 2022 Mar 3;13:823182. doi: 10.3389/fgene.2022.823182 (PMC8927288; doi:10.3389/fgene.2022.823182)
Supplement: Supplementary file 1 [file Presentation1.PPTX]

## Slide 1
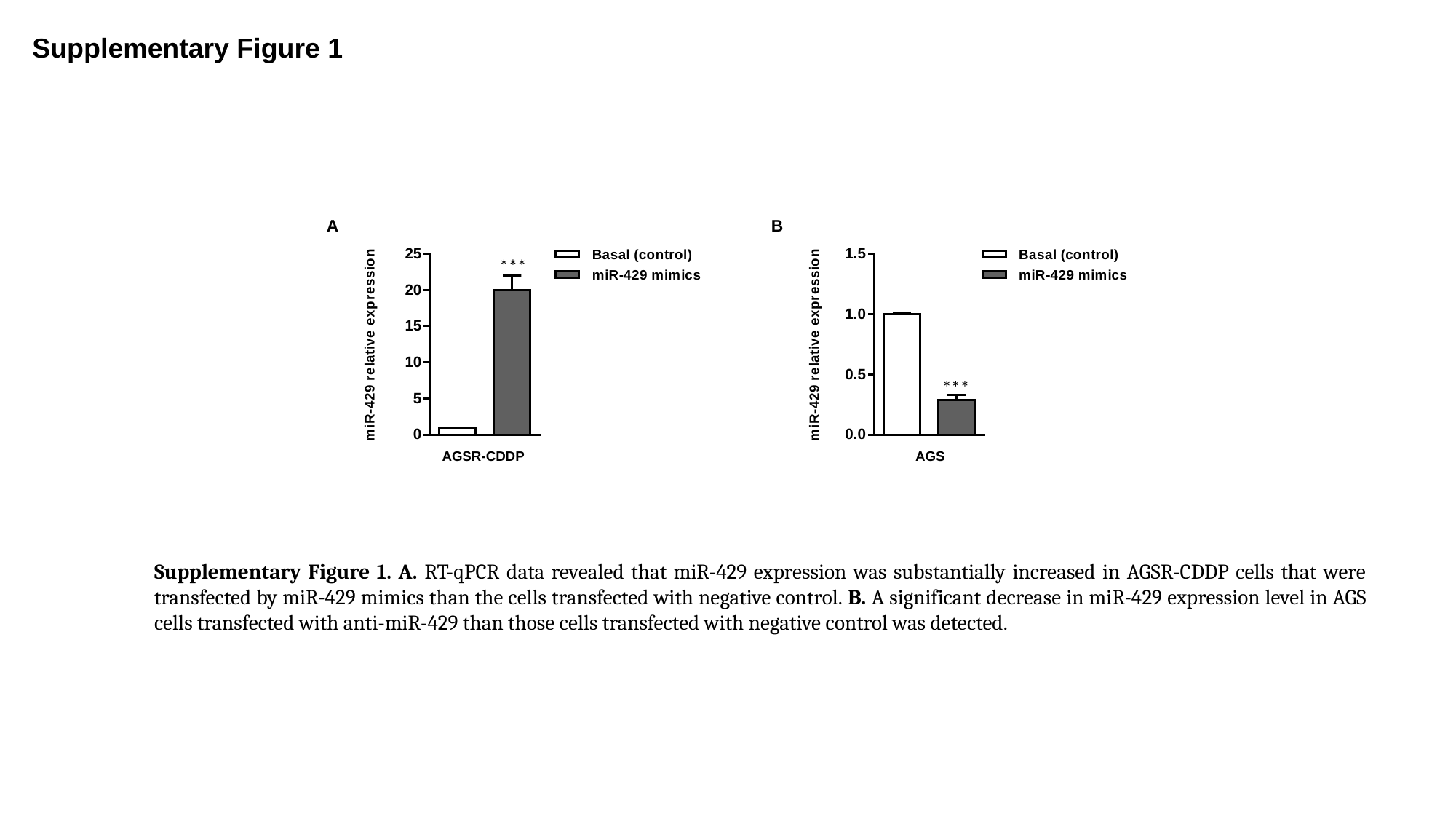

Supplementary Figure 1
A
AGSR-CDDP
***
B
AGS
***
Supplementary Figure 1. A. RT-qPCR data revealed that miR-429 expression was substantially increased in AGSR-CDDP cells that were transfected by miR-429 mimics than the cells transfected with negative control. B. A significant decrease in miR-429 expression level in AGS cells transfected with anti-miR-429 than those cells transfected with negative control was detected.

## Slide 2
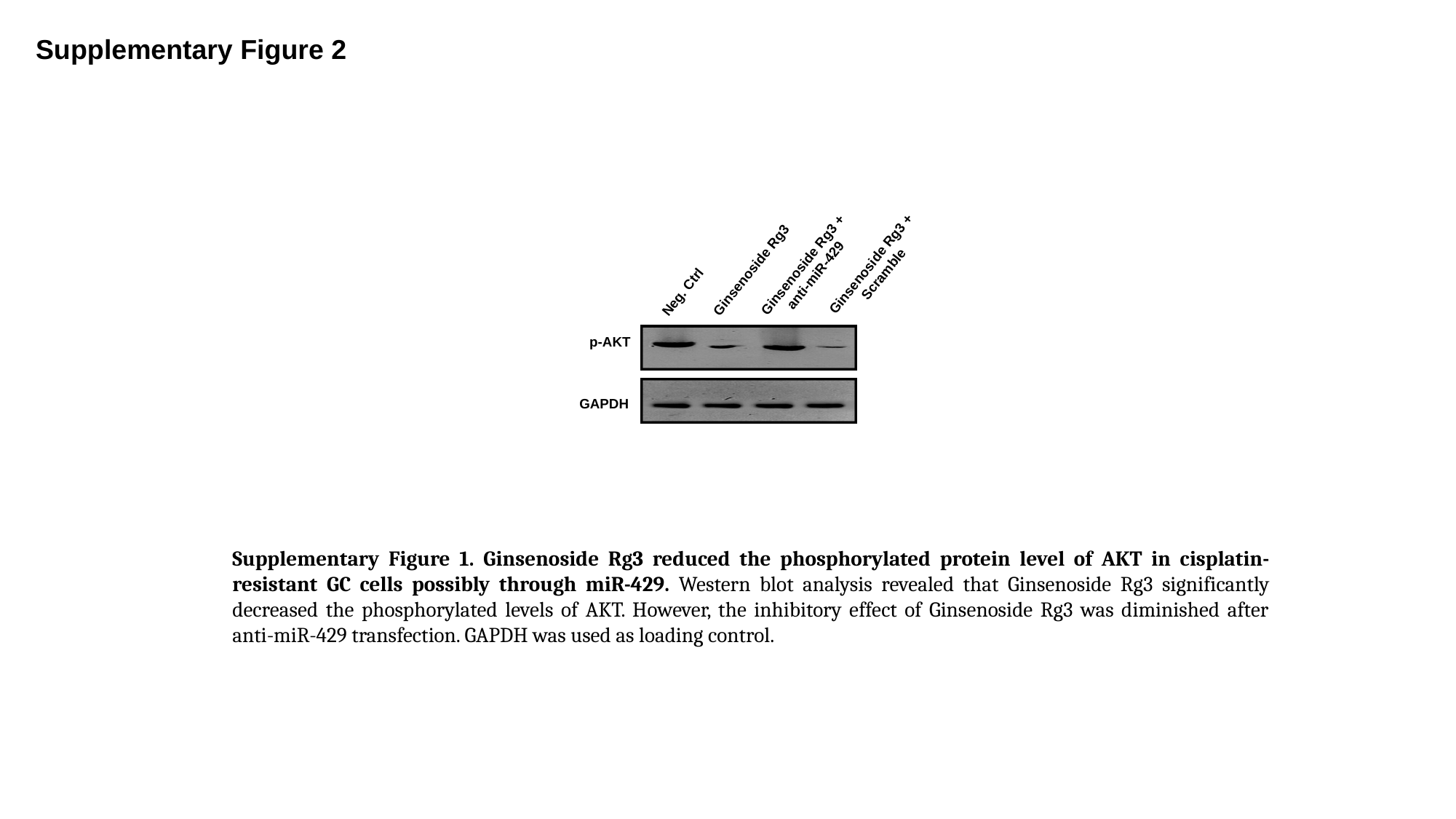

Supplementary Figure 2
Ginsenoside Rg3 + Scramble
Ginsenoside Rg3 + anti-miR-429
Ginsenoside Rg3
Neg. Ctrl
p-AKT
GAPDH
Supplementary Figure 1. Ginsenoside Rg3 reduced the phosphorylated protein level of AKT in cisplatin-resistant GC cells possibly through miR-429. Western blot analysis revealed that Ginsenoside Rg3 significantly decreased the phosphorylated levels of AKT. However, the inhibitory effect of Ginsenoside Rg3 was diminished after anti-miR-429 transfection. GAPDH was used as loading control.
