# Supplementary figures and images for: Ginsenoside Rg3 Alleviates Cisplatin Resistance of Gastric Cancer Cells Through Inhibiting SOX2 and the PI3K/Akt/mTOR Signaling Axis by Up-Regulating miR-429
Source: Front Genet. 2022 Mar 3;13:823182. doi: 10.3389/fgene.2022.823182 (PMC8927288; doi:10.3389/fgene.2022.823182)

## Slide 1
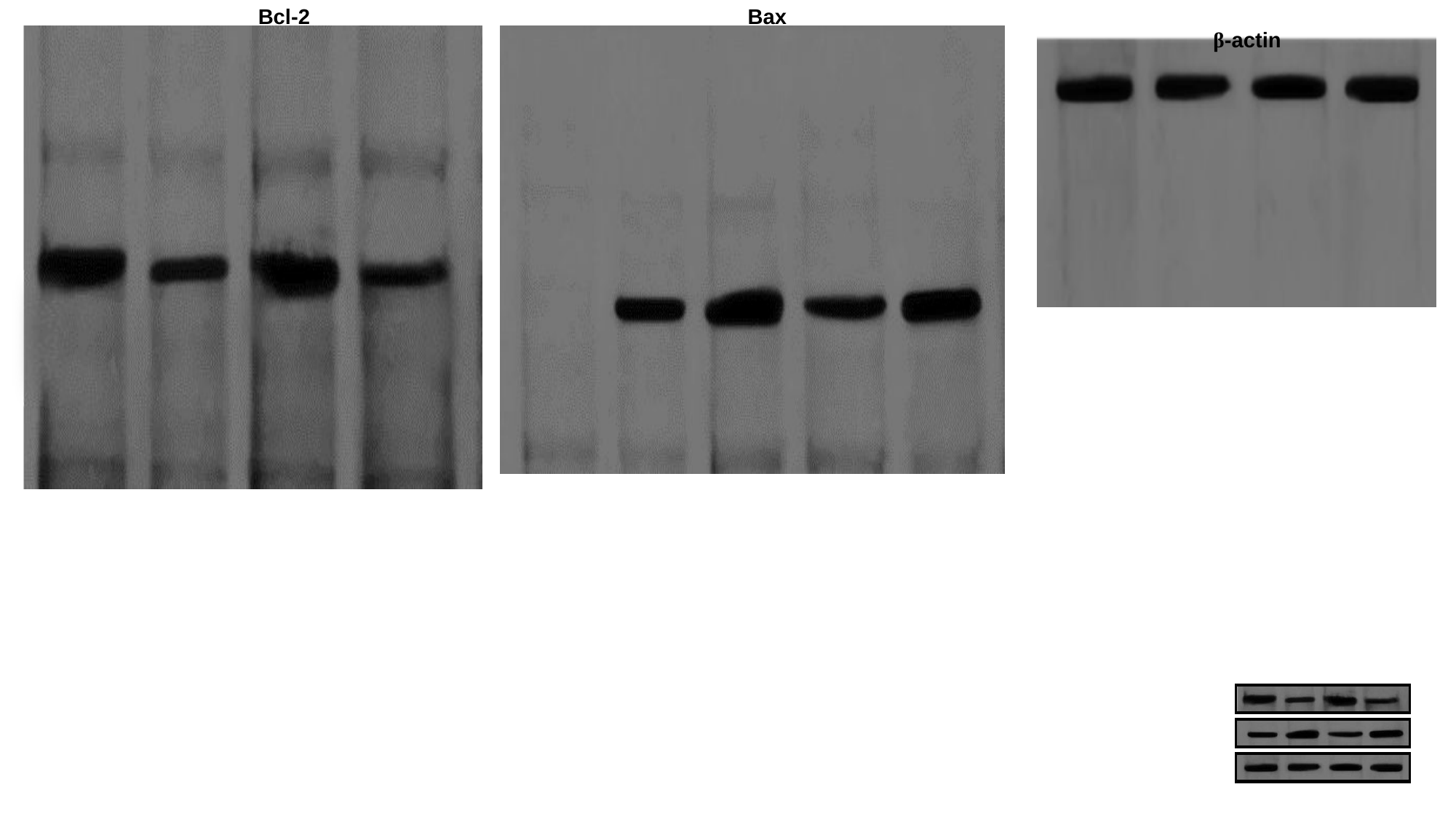

Bcl-2
Bax
β-actin

## Slide 2
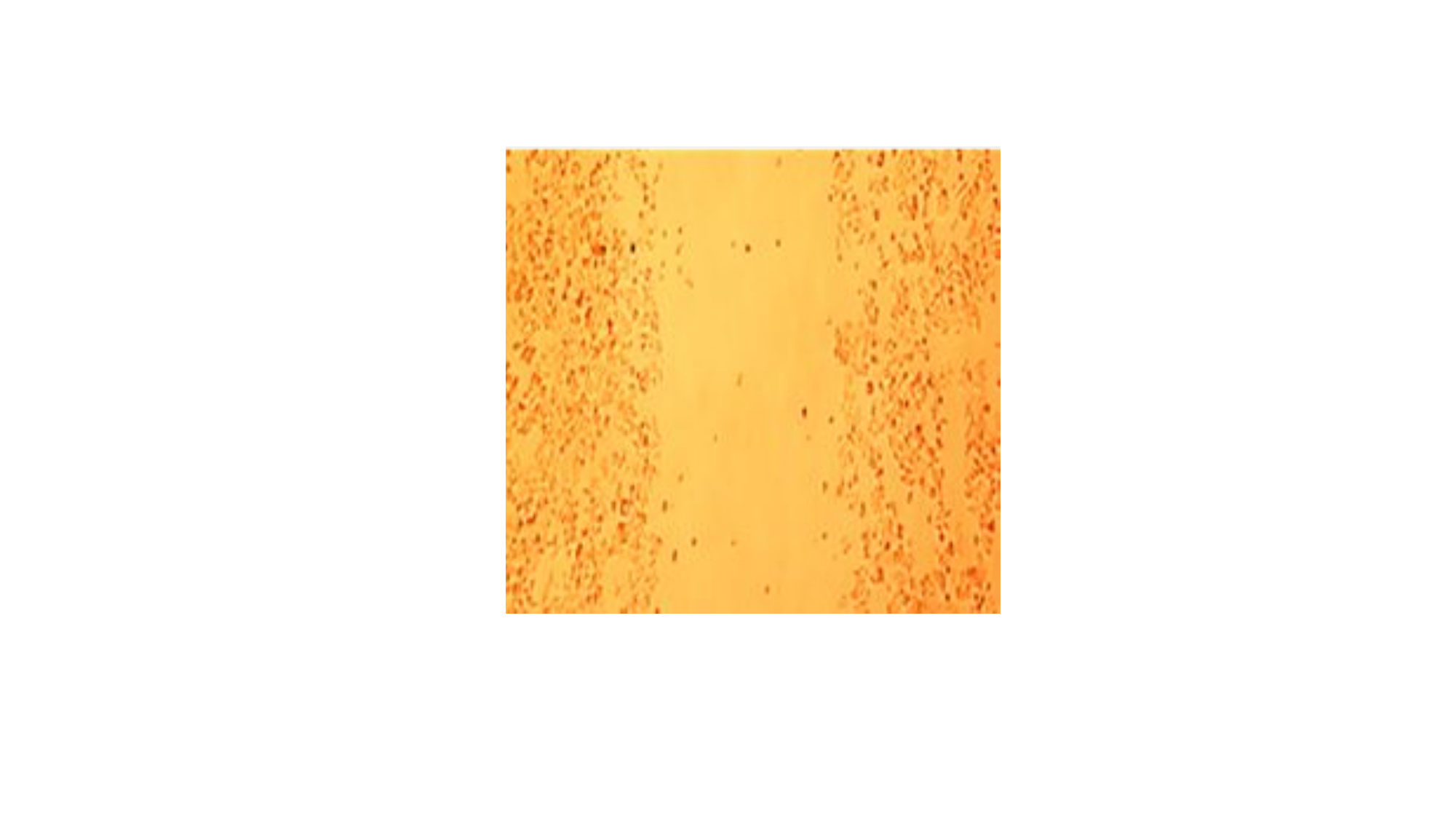

## Slide 3
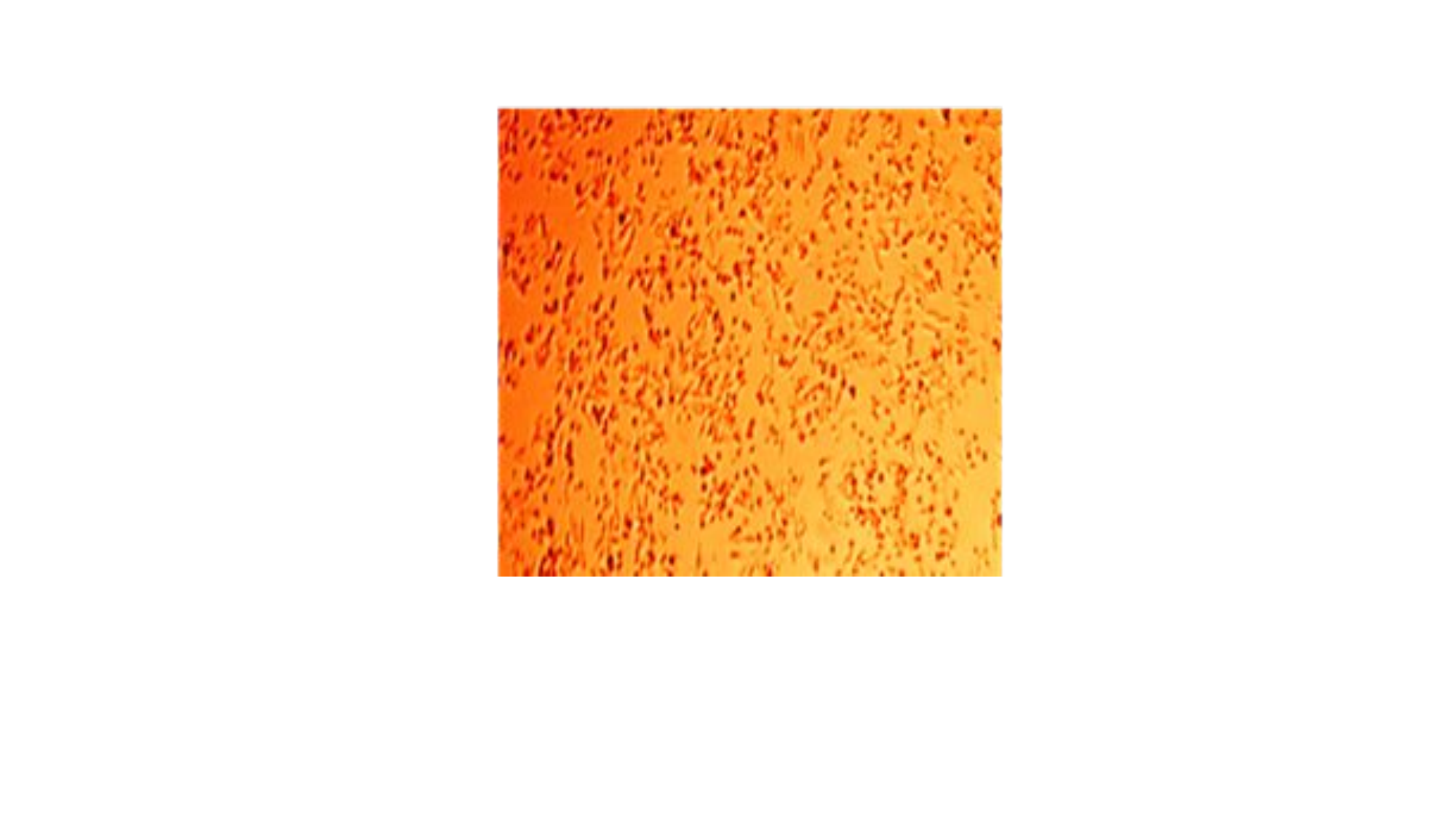

## Slide 4
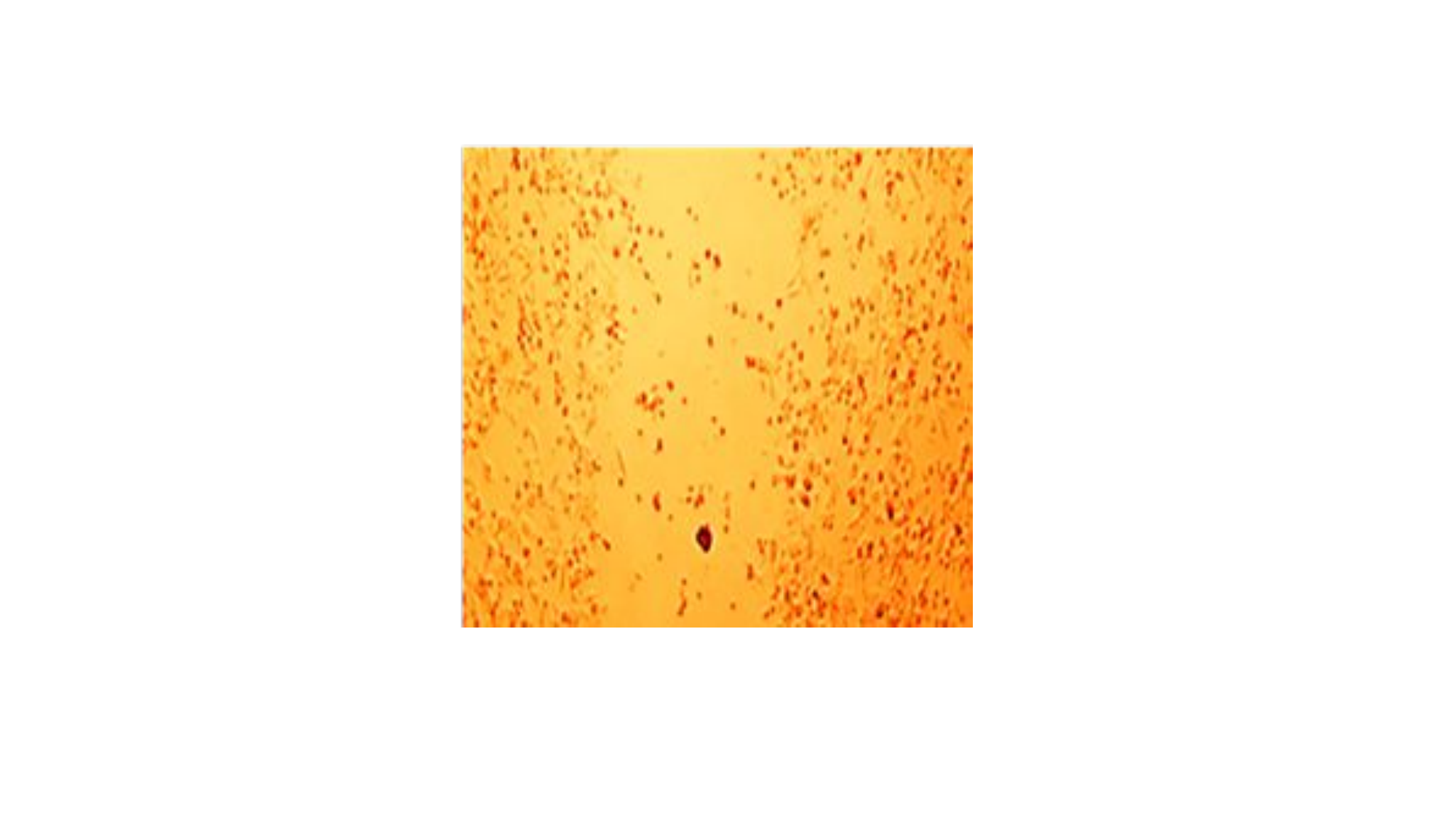

## Slide 5
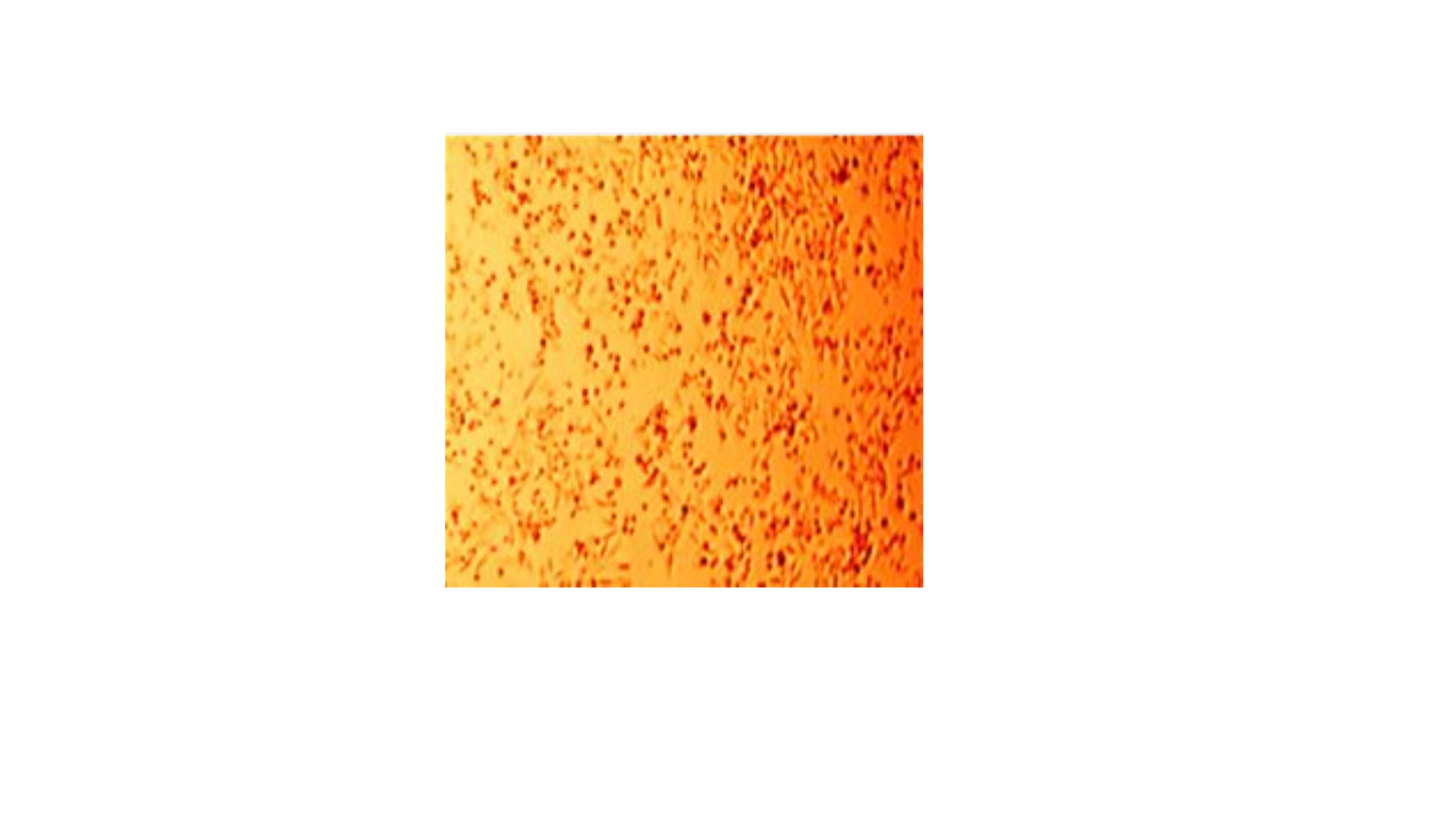

Supplement: Supplementary file 2 [file DataSheet1.ZIP › Full gel and Photo.pptx]
